# Antimicrobial Susceptibility Profile of Pathogenic and Commensal Bacteria Recovered from Cattle and Goat Farms

**DOI:** 10.3390/antibiotics12020420

**Published:** 2023-02-20

**Authors:** Winnie Mukuna, Tobenna Aniume, Bharat Pokharel, Collins Khwatenge, Ashesh Basnet, Agnes Kilonzo-Nthenge

**Affiliations:** 1 Department of Agriculture and Environmental Sciences, Tennessee State University, 3500 John A. Merritt Boulevard, Nashville, TN 37209, USA; 2 Department of Human Sciences, Tennessee State University, 3500 John A. Merritt Boulevard, Nashville, TN 37209, USA

**Keywords:** antimicrobial resistance, Enterobacteriaceae, cattle and goat farms, raw milk

## Abstract

The use of antibiotics in food animals results to antimicrobial resistant bacteria that complicates the ability to treat infections. The purpose of this study was to investigate the prevalence of pathogenic and commensal bacteria in soil, water, manure, and milk from cattle and goat farms. A total of 285 environmental and 81 milk samples were analyzed for Enterobacteriaceae by using biochemical and PCR techniques. Susceptibility to antibiotics was determined by the Kirby–Bauer disk diffusion technique. A total of 15 different Enterobacteriaceae species were identified from goat and cattle farms. Manure had significantly higher (*p* < 0.05) Enterobacteriaceae (52.0%) than soil (37.2%), trough water (5.4%), and runoff water (5.4%). There was a significant difference (*p* < 0.05) in *Enterobacteriaceae* in goat milk (53.9%) and cow milk (46.2%). *Enterobacteriaceae* from environment showed 100% resistance to novobiocin, erythromycin, and vancomycin *E. coli* O157:H7, *Salmonella* spp., *Enterococcus* spp., and *Listeria monocytogenes* displayed three, five, six, and ten. AMR patterns, respectively. NOV-TET-ERY-VAN was the most common phenotype observed in all isolates. Our study suggest that cattle and goat farms are reservoirs of multidrug-resistant bacteria. Food animal producers should be informed on the prudent use of antimicrobials, good agricultural practices, and biosecurity measures.

## 1. Introduction

Antimicrobial resistance (AMR) is one of the most reported health challenges, and associated deaths could rise to 10 million by 2050 [1]. Antibiotics are indispensable in treating bacterial diseases in both humans and animals. They are prevailing remedies that are useful to combat infections; however, the rising AMR is compromising their efficacy. According to Habboush and Guzman [2], antibiotic resistance arises when bacteria evolve and develop multiple different mechanisms to escape the effectiveness of antibiotics. It is documented that antibiotics use in food animal production is a foremost cause of the evolving AMR in humans [3]. Antimicrobials are commonly used in livestock for prevention and control of diseases [4], as well as for sustainable production [5]. According to Boeckel et al. [6], in 2013, 131,109 tons of antimicrobials globally were used in food animals and anticipates escalating to 200,235 tons by 2030. In the USA, more than half of the 14,000 tons of antimicrobials traded in 2016 were used in animal agriculture [7]. Specifically, antimicrobials in dairy cattle production are commonly used to control and treat clinical and subclinical mastitis, which leads to a large economic loss worldwide [8]. Treatment of sick farm animals should not be evaded or deferred as it can result to animal death, suffering, and economic losses. According to Kasimanickam et al. [9], antibiotics contribute to good animals’ health, well-being, and food safety, as well as the improvement of the livelihoods of growers. However, antimicrobial use contributes to agricultural AMR [10] and creates an environment that selects for the expression and exchange of antimicrobial resistance genes (ARGs) in both commensal and pathogenic bacteria [11]. 

In livestock farming, animals expel antibiotic-resistant bacteria (ARB) in their gastrointestinal track; consequently, ARGs are spread into receiving environment including soil and waterbodies [3]. Contaminated soils and water bodies harbor resistant pathogens and resistant genes that may enter the food chain [12], hence a potential pathway transfer of ARB to humans. The use and overuse of antibiotics in food animals has led to antimicrobial resistant ARB and ARGs in our environment. Consequently, ARB and ARGs shift to humans via direct interface with animals, exposure to animal waste, and consumption of contaminated foods of animal origin and fresh produce [13,14]. Transfer of AMR from animals to humans and the environment is not only limited to foodborne pathogens, but also to commensal bacteria as well. AMR *Enterococcus* spp. and other commensal bacteria have been isolated from manure and soil in dairy farms [15]. Consumers, through ingestion of tainted animal food commodities, may be exposed to ARB and ARGs [16]. 

AMR is a challenge with imperative magnitude in the farming environment that contribute to its progression and spread. A lot of studies have focused on the incidence of ARB in aquatic environments; however, there are still gaps, particularly those associated with agriculture [17]. Monitoring of AMR in diverse animal agriculture could offer essential data for mitigating the spread of ARB in our environment. Thus, this study aims to identify and characterize the phenotypic AMR profiles of both pathogenic and commensal bacteria isolated from environmental samples and raw milk collected from small dairy cattle and goat farms in Nashville, Tennessee. 

## 2. Results

### 2.1. Enterobacteriaceae in Cattle and Goat Farms

Out of 285 environmental (manure, soil, water) samples, 148 isolates were identified as Enterobacteriaceae at the ≥ 90% confidence level (Table 1). A total of 15 different Enterobacteriaceae species were identified from goat and cattle farms (Table 1). Overall, *E. coli* prevalence (76.4%) was significantly higher (*p* < 0.05) than *Enterobacter cloacae* (10.1%), *Serratia marcascens* (3.4%), *Enterobacter aerogenes* (2.0%), and *Serratia odorifera* (1.4%). Our results demonstrate that manure had significantly higher (*p* < 0.05) Enterobacteriaceae (52.0%; 77/148) than soil (37.2%; 55/148), trough water (5.4%; 8/148), and runoff water (5.4%; 8/148). 

Table 1 shows that *E. coli* isolates were displayed highest in manure (45.9%; 68/148), followed by soil (23.6%; 35/148), runoff water (4.7%; 7/148), and trough water (2%; 3/148). Specifically, 16.9% (25/148), 15.5% (23/148), and 13.5% (20/148) of *E. coli* isolates occurred in manure from CF1, GF, CF2, respectively. *Yersinia enterocoliticas* (0.7%) was only isolated from runoff water in goat farm. 

### 2.2. Enterobacteriaceae in Cattle and Goat Raw Milkidentified as Having a Bacteria of the Enterobacteriaceae 

Enterobacteriaceae strains were recovered from cow and goat raw milk. This demonstrated that, out of 81 milk samples, only 16.0% (13/81) bacterial species were identified, as indicated in Table 2. By comparison, there was no significant difference (*p* < 0.05) in the percentage of Enterobacteriaceae in cow milk (46.2%) and goat milk (53.8%). The most prevalent Enterobacteriaceae species was *Pantoea* spp. 4 at 38.5% (5/13) in cow milk, although it was not significantly higher (*p* < 0.05) than other isolates (Table 2). *E. coli* was the second most common Enterobacteriaceae species at 23.1% (3/13) and was only present in goat milk. According to our results, only 23.1% (3/13) of identified spices were *E. coli*.

### 2.3. Prevalence of Pathogenic Bacteria in Cattle and Goat Farm Environments

**Figure 1:**
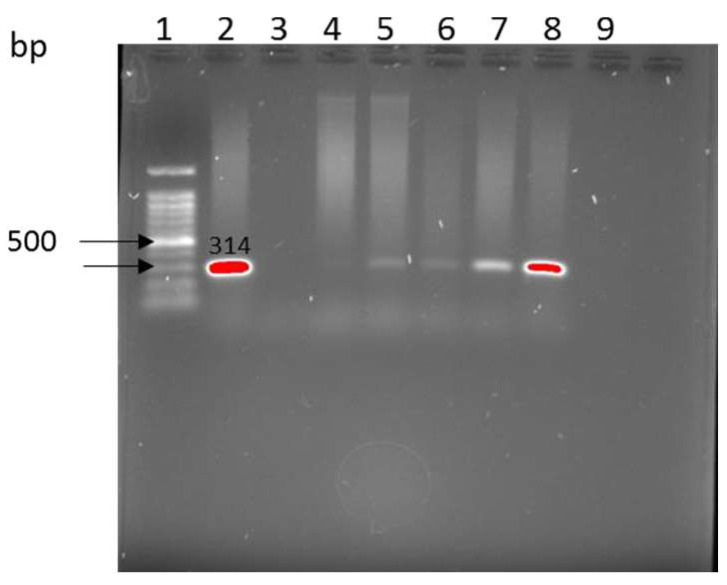
Amplification of the *rfbE* gene in *E. coli* O157:H7. Lane 1: 1 kb ladder, lane 2: positive control *E. coli* O157:H7 ATTC, Lane 3: water negative control, Lane 4–8: samples.

Presumptive pathogenic bacteria were confirmed by PCR as described in Materials and Methods. Our results showed that *rfbE* gene (Figure 1) were amplified in *E. coli* O157:H7 from cattle farms. 

*E. coli* O157:H7, *Salmonella* spp., *Listeria monocytogenes*, and *Enterococcus* spp. isolated from cattle and goat farms are presented in Table 3. 

**Figure 2:**
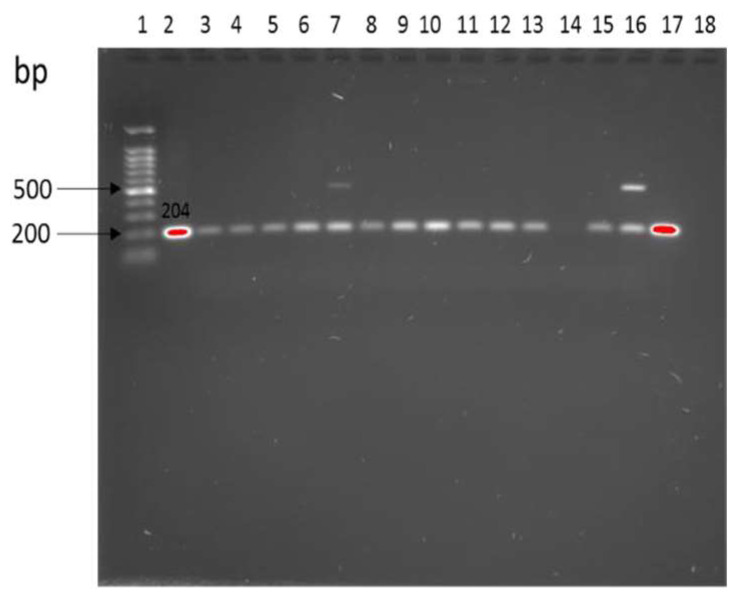
Amplification of the *ompC* gene in *Salmonella* spp. Lane 1: 1 kb ladder, lane 2: positive control *Salmonella typhimurium* ATTC 13311, lane 3–17: samples, lane 18: *E. coli* ATTC 25922 negative controls.

Overall, about 1.9% (5/285) of environmental samples (water, manure, water) from cattle and goat farms were positive for *E. coli* O157:H7. Precisely, *E. coli* O157:H7 was detected in manure at 0.7% (2/285), soil at 0.4% (1/285), and runoff water at 0.4% (1/285) from CF1, and in manure, at 0.4% (1/285) from CF2 (Table 3). Notably, no *E. coli* O157:H7 was detected in goat farm (GF). Generally, in CF1, *Salmonella* spp. was isolated from the environment at 10.4%. *Salmonella* spp. was identified at 0.7%, 1.1%, and 0.4% in manure, soil, and runoff water, respectively. Approximately, 0.7%, 2.8%, and 0.4% of *Salmonella* spp. isolates were detected in manure, soil, and runoff water in CF2, respectively. In GF, 2.1%, 1.8%, and 0.4% *Salmonella* spp. was isolated from manure, soil, and trough water, respectively *Salmonella* spp. was confirmed by amplification of targeted *ompC* gene (Figure 2) by PCR.

**Figure 3:**
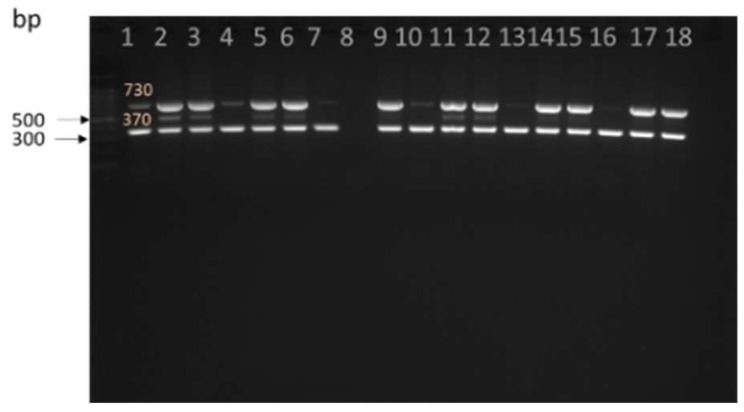
Amplification of *prs* for *Listeria* and *hly* of genes in *L. monocytogenes*. Lane 1 and 9 positive control: *Listeria monocytogenes* ATCC 19115, lane 8- Blank/control, Lane 2–7 and 10–18 positive samples.

The *hly* and *prs* genes for *L. monocytogenes* and *Listeria* spp, respectively, were amplified as demonstrated in Figure 3. Overall, about 23.2% (66/285) of environmental samples (water, manure, water) from cattle and goat farms were positive for both *L. monocytogenes* and *Listeria* spp. (Table 3). The highest occurrence of *L. monocytogenes* at 3.9% (11/285) was observed in soil (CF1) and manure CF2 and was not significantly different (*p* < 0.05) from soil at 3.2 (9/285) in goat farm. *L. monocytogenes* was detected at 0.4% (1/285) in both trough water and runoff water.

**Figure 4:**
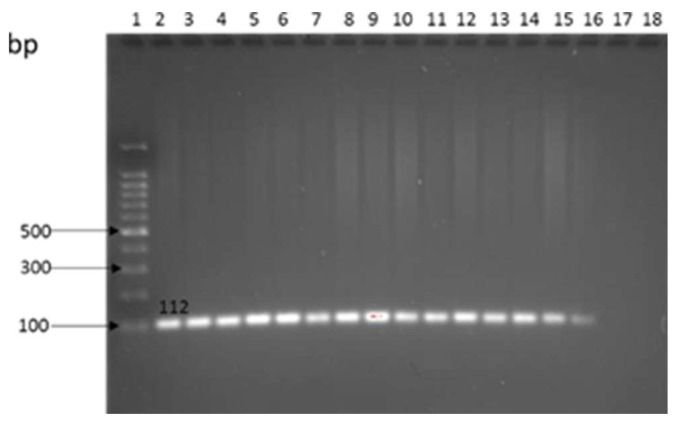
Amplification of the *Tuf* gene in *Enterococcus* spp. Lane 1: 1 kb ladder, lane 2: *Enterococcus faecium* ATCC 35667 positive control, lane 3–17: samples, lane 18: water negative control.

The amplification of the *Tuf* gene confirmed the prevalence of *Enterococcus* spp. in environmental samples in cattle and goat farms (Figure 4). *Enterococcus* spp. at 24.2% (66/285) was the most common pathogen isolated from farms. The highest occurrence of *Enterococcus* spp. at 3.2% (9/285) was observed in soil at CF1 and was not significantly different (*p* < 0.05) from manure at 3.5% (10/285) in CF2. *Enterococcus* spp. was also detected in both trough water and runoff water, as displayed in Table 3.

### 2.4. Antibiotic Resistant Enterobacteriaceae and Phenotype Patterns

**Figure 5:**
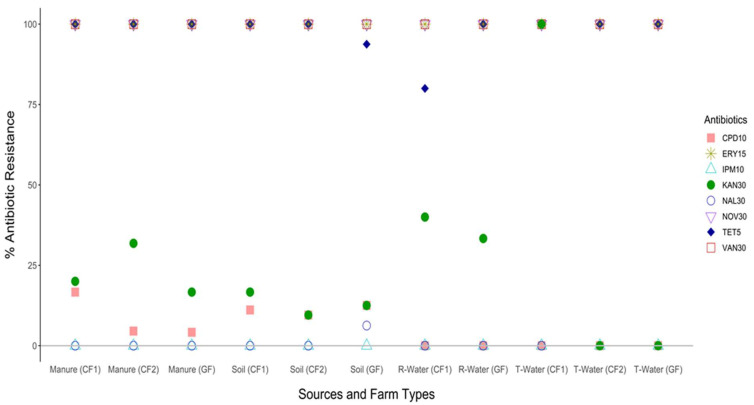
Percentage (%) resistance of Enterobacteriaceae isolated from different farm environment in cattle and goat farms. CF1: Cattle Farm 1; CF2: Cattle Farm 2; GF: Goat Farm.

Enterobacteriaceae from environment showed 100% resistance to novobiocin, erythromycin, and vancomycin. Resistance to tetracycline ranged between 75% and 100%. Notably, Enterobacteriaceae isolates displayed low resistance (≤25%) to cefpodoxime and nalidixic acid. Most of the Enterobacteriaceae isolates were susceptible to imipenem (Figure 5).

Antibiotic resistance phenotypes of Enterobacteriaceae isolates are shown in Table 4. Generally, Enterobacteriaceae isolates showed a total of four antibiotic resistant patterns: NOV-TET-ERY-VAN, NOV-ERY-VAN, NOV-TET-ERY-VAN-KAN, and NAL-NOV-TET-ERY-VAN. *E. coli* isolates displayed three antibiotic resistant patterns: NOV-TET-ERY-VAN (*n* = 87; CF1 = 30, CF2 = 32, GF = 25), NOV-ERY-VAN (*n* = 25; CF1 = 12, CF2 = 5, and GF = 8), and NOV-TET-ERY-VAN-KAN (*n* = 1; CF1 = 1). Notably, *Yersinia enterocolitica* displayed one resistant pattern: NOV-ERY-VAN (CF1 = 1).

**Figure 6:**
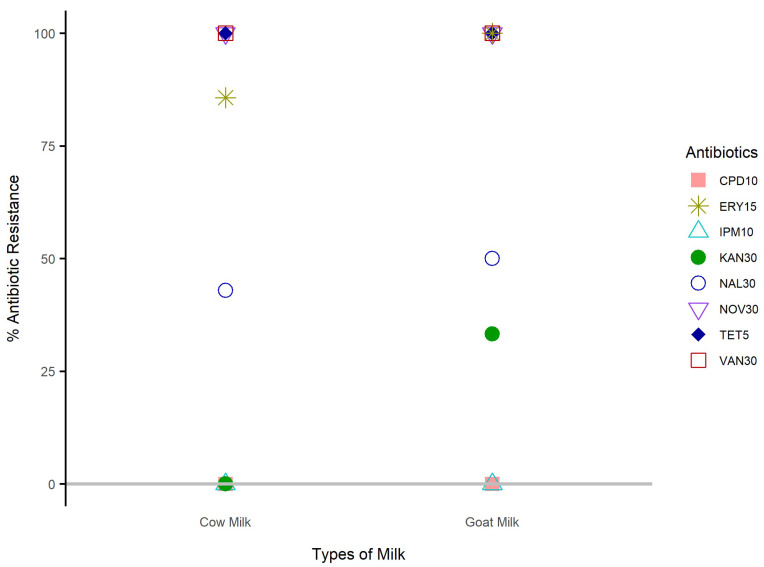
Percentage (%) resistance of Enterobacteriaceae isolates from cow and goat raw milk. CF1:Cattlle Farm 1; CF2:Cattlle Farm 2; GF: Goat Farm.

Enterobacteriaceae isolated from cow and goat raw milk was 100% resistant to tetracycline, vancomycin, and novobiocin. Generally, erythromycin resistance was above 75% for isolates from both cow and goat milk. Enterobacteriaceae isolates from cow milk were susceptible to cefpodoxime, kanamycin, and imipenem (Figure 6). Table 5 shows six different AMR patterns of Enterobacteriaceae, and the most common pattern was NOV-TET-ERY-VAN (*n* = 4), followed by NOV-TET-VAN (*n* = 2), and NAL-NOV-ERY-VAN, NOV-ERY-VAN, NOV-VAN (*n* = 1), and TET-VAN (*n* = 1). *E. coli* and *Pantoea* spp. 4 displayed TET-VAN and NOV-VAN patterns, respectively.

### 2.5. Multi-Drug Resistance of Pathogenic Bacteria in Cattle and Goat Farms

A number of forty-three (*n* = 43) pathogeic isolates were selected and tested for multi-drug resistance. All pathogenic isolates showed resistance to seven or eight antibiotics, as shown in Table 6. The overall percentage resistance was significantly higher (*p*< 0.05) for vancomycin (83.7%), novobiocin (79.1%), and erythromycin (72.1%) as compared to tetracycline (48.8%), cefpodoxime (41.9%), kanamycin (37.2%), and nalidixic acid (37.2%). Antimicrobial sensitivity demonstrated that *E. coli* O157:H7 and *Salmonella* spp. were resistant to seven out of the eight antbiotics. All *Enterococcus* spp. and *L. monocytogenes* isolates were resistant to all eight antibiotics.

Our results (Table 6) show that *E. coli* O157:H7 demonstrated higher resistance to vancomycin, (7.0%) and erythromycin (7.0%) than to tetracycline (4.7%), novobiocin (4.7%), and nalidixic acid (4.7%), although it was not significantly different (*p* < 0.05). Notably, all *E. coli* O157:H7 isolate were susceptible (100%) to imipenem. 

*Salmonella* spp. resistance was significantly higher (*p* < 0.05) to vancomycin (27.9%), novobiocin (27.9%), and erythromycin (27.9%), as compared to tetracycline (11.6%), cefpodoxime 7.0%), kanamycin (2.3%), and nalidixic acid (2.3%). *Salmonella* spp. did not display any resistance to imipenem.

Statistical analysis showed that *Enterococcus* spp. isolates (Table 6) displayed significantly higher (*p* < 0.05) resistance to vancomycin (32.6%), novobiocin (30.2%), and erythromycin (30.2%), as compared to tetracycline (7.0%), cefpodoxime (7.0%), imipenem (4.7%), kanamycin (4.7%), and nalidixic acid (4.7%). 

*Listeria monocytogenes* isolates displayed significantly higher (*p* < 0.05) resistance for cefpodoxime (27.9%), kanamycin at (27.9%), tetracycline (25.6%), and nalidixic acid (25.6%), as compared to vancomycin (16.3%) and novobiocin (16.3 %). Furthermore, significantly lower (*p* < 0.05) amounts of resistance were detected in erythromycin (7.0%) and imipenem (2.3%).

Antibiotic resistance phenotypic patterns of the retrieved bacterial pathogens from cattle and goat farms were characterized for their antibiotic resistance phenotypes (Table 7). Intermediate phenotypes of AMR were excluded from this analysis. *E. coli* O157:H7 isolates from CF1 presented three AMR patterns: VAN-NOV-KAN-ERY-NAL, TET-VAN-ERY-NAL, and TET-VAN-CEF-NOV-ERY.

The five different phenotypic patterns observed in *Salmonella* spp. were as follows: VAN-NOV-ERY, VAN-CEF-NOV-ERY, TET-VAN-CEF-NO-ERY, TET-VAN-CEF-NOV-KAN-ERY-NAL, and TET-VAN-NOV-ERY. VAN-NOV-ERY was the most frequent pattern observed among *Salmonella* spp. isolates. *Enterococcus* spp. presented six AMR, with VAN-NOV-ERY pattern occurring the most among the isolates. Our findings reveal that *Listeria monocytogenes* isolates exhibited the most multidrug-resistant patterns (*n* = 10). Notably, TET-CEF-KAN-NAL pattern was displayed in both cattle and goat farms.

## 3. Discussion

### 3.1. Enterobacteriaceae in Manure, Soil, and Water in Cattle and Goat Farms

Our results and those of previous studies indicate that animal farms harbor some associates of Enterobacteriaceae family that are foodborne pathogens [18]. Although other Enterobacteriaceae species were characterized in the current study, emphases were on *E. coli* as it is a more specific indicator of fecal contamination than other coliforms [19]. Overall, our results demonstrated that *Escherichia coli* isolates were found most in manure (45.9%), followed by soil (23.6%), runoff water (4.7%), and trough water (2%). *E. coli* is extensively found in the guts of animals as commensal microorganism [20], and ruminants including cattle are considered as the major reservoirs [21]. According to Kulow et al. [22], *E. coli* in manure is attributed to the cattle intermittent shedding into fecal matter [23]. Significantly lower rates for other important Enterobacteriaceae, including *Enterobacter cloacae*, *Escherichia fergusonii*, and *Klebsiella pneumoniae*, were identified in manure, soil, and water in cattle and goat farms. Our findings agree with Davin–Regli and Pages [24] that *Enterobacter cloacae* resides in water, soil, and manure in agricultural lands. Although not commonly associated with foodborne diseases, *Enterobacter cloacae* is a widely known nosocomial pathogen and third most causative bacteria in hospital acquired infections after *E. coli* and *Klebsiella pneumoniae* [25]. In our study, *Escherichia fergusonii* demonstrated a low prevalence in manure from cattle farm (CF1), this bacterium has been reported in farm animals [26]. *E. fergusonii* is documented to cause severe pneumonia and death in adult cows [27]. Since *E. fergusonii* reside in foods of animal origin, it has a potential risk to food safety and public health [28]. 

### 3.2. Enterobacteriaceae in Raw Cow and Goat Milk

This study showed that Enterobacteriaceae species, such *E. coli*, *Pantoea* spp., *Enterobacter* spp., *Escherichia hermannii*, and *Klebsiella pneumoniae,* were present in cow and goat raw milk. Approximately 23.1% of goat milk samples were positive for *E. coli*, as was the case in a previous study [29]. It is possible that goat milk may have been contaminated during the milking process. *E. coli* was not present in cow milk; however, Samet–Bali et al. [30] and Saba et al. [31] reported higher incidences in cow milk at 32.5% and 49.3%, respectively. *E. coli* is a naturally occurring microorganism in the guts of humans and animals [20] and is used as indicator of fecal contamination in food and water safety microbiological analysis [32]. *E. coli* are commensal bacteria; however, pathogenic *E. coli* can result in zoonotic illness that positions as a public health risk. *Pantoea* spp., which was displayed in cow milk, is reported to be a naturally occurring organism in the environment and agricultural settings [33]. It is an opportunistic pathogen that causes bacteremia in immunocompromised individuals [34]. The presence of *Pantoea* spp. in cow milk is a concern, especially if consumed raw, as it is a health risk. Data from this study suggest that raw milk has the potential to carry potentially pathogenic microorganisms, and thus cow and goat milk should not be consumed raw.

### 3.3. Occurrence of Pathogenic Bacteria in Cattle and Goat Farms

Notably, our findings showed that it is important when detecting pathogenic bacteria from farming environment to enrich environmental samples (manure, soil, water) with recommended supplements. In this study, pathogenic bacteria were only detected when enrichment supplement specific for each bacterium were used. *E. coli* O157:H7 was present in manure (0.7%), soil (0.4%), and runoff water (0.4%) in cattle farm (CF1). In CF2, *E. coli* O157:H7 was only isolated from manure (0.4%). Our study agrees with previous studies that *E. coli* O157:H7 is present in cattle manure [35,36]. Notably, *E. coli* O157:H7 was not present in trough water; however, it was present in runoff water. This pathogen is zoonotic and is carried by cattle in their gastrointestinal tracts [37]. According to Chase-Topping et al. [38], high levels of shedding by cattle account for most *E. coli* O157:H7 in the environmental contamination. *E. coli* O157:H7 dispersion from manure/animal feces into soils and runoff water represents a human health concern. Notably, *E. coli O157:H7* was not present in goat farm (GF). Although *E. coli* O157:H7 was not detected from manure in goat farm in our study, this pathogen was isolated from goat feces (11.1%) at a USDA-inspected processing plant in the southeastern United States [39]. It is a public health risk when *E. coli* O157:H7 diffuses from manure amended soils to neighboring rivers and streams through water runoff water [40]. Irrigation of fresh produce with surface water contaminated with *E. coli* O157:H7 poses a great risk to consumers, since most fresh produce is consumed raw. *Escherichia coli* O157:H7 has a zero tolerance in food products due to its low infectious dose. *E. coli* O157:H7 infections may also occur due to direct interactions with animals or contaminated food products of animal origin [41]. Although several actions are taken during food processing, consumers may not be protected from this pathogen [42]. Animal handlers in dairy production systems should take extra thoughtfulness when handling livestock, since it is a potential route of infection with *E. coli* O157:H7. 

Our results showed more prevalence of *Salmonella* spp. (10.4%) than *E. coli* O157:H7 (1.9%) in the farm environment. *Salmonella* spp. was detected in all farms and was present in feces, soil, trough water, and runoff water. Our findings agree with Sobur et al. [43] that *Salmonella* spp. was more prevalent in soil than in water. Although our findings show lower *Salmonella* spp. (2.1%) occurrence in goats’ feces, it agrees with previous studies that demonstrated the occurrence of the pathogen at 3. 7% and 3. 4% in the United States [44] and Ethiopia [45], respectively. *Salmonella* spp. can diffuse via feces from infected livestock to their surrounding environment including soil and water bodies. According to Huston et al., [46], *Salmonella* spp. can persevere in the farm settings for up to six years in animal feces. The main risk for zoonotic salmonellosis from cattle is exposure to contaminated meat through fecal contamination of the carcass during slaughter [47].

In the present study, 23.4% of environmental samples were positive for *Listeria monocytogenes.* This pathogen occurred in all farms and was most prevalent in soil, followed by manure, trough water, and runoff water. According to Vijayakumar and Muriana [48], this pathogen often occurs in the farm environment including faces, manure, soil, and water sources through which it penetrates the food chain. According to Borucki et al., [49] and Mohammed et al. [50], dairy farming environment is considered an important reservoir of *Listeria Monocytogenes*, which may be transferred to animal food products, causing listeriosis [51]. *Listeria* spp. in animal feces may also be transferred to crops through water used for irrigation and application of manure into agricultural soils [52], hence it is a major concern in public health.

The study found that *Enterococcus* spp. was the most prevalent pathogen at 24.5% and was isolated from manure, soil, water trough water, and runoff water. *Enterococci* spp. are ubiquitous organisms that are extensively detected in bovine feces, soil, water, plants, and the gastrointestinal tracts (GI) of humans and animals [53,54]. According to Fang, [55], *Enterococcus* spp. is an emerging pathogen that is linked to foodborne illness and cause various infections including nosocomial infections. This pathogen has been used as pointers of microbiological quality of fresh produce [56] and their presence in water as an indication of fecal contamination [57]. The presence of *Enterococcus* in cattle and goat farms is a suggestion that the dairy production systems are reservoirs of this pathogen.

Overall, our data and other previous studies demonstrate that manure, soil, and water are important sources of *Escherichia coli* O157:H7, *Salmonella* spp., *L. monocytogenes*, and *Enterococcus* spp. [58,59,60,61]. Occurrence of *E. coli* O157:H7 and *Salmonella* spp. in dairy farms have been documented [62,63]. Although *E. coli* O157:H7, *Salmonella* spp., and *L. monocytogenes* were not isolated from raw milk in our study, they have been associated with the consumption of raw milk from cows and goats [64,65]. Nevertheless, pathogenic bacteria may contaminate raw milk via fecal contamination by excretion into the milk.

### 3.4. Antibiotic Resistance in Enterobacteriaceae

According to our findings, phenotypic screening of antimicrobial resistance among Enterobacteriaceae from cattle and goat farms displayed multi-drug resistance to indispensable antibiotics in both human and animal medicine. Enterobacteriaceae have been associated with higher mortality than other microbes [66]. Our results showed that all Enterobacteriaceae from soil, manure, and water in cattle and goat farms was highly resistant to novobiocin (100%), erythromycin (100%), and vancomycin (100%). Enterobacteriaceae isolates from runoff water in goat farm and trough water in cattle farm (CF2) were 100% resistant to tetracycline. Kanamycin resistance in all Enterobacteriaceae isolates ranged from 0 to approximately 33.3%. Generally, cefpodoxime and nalidixic acid showed relatively low resistance ranging from 0 to 16.7%. Notably, all Enterobacteriaceae isolates from farm environment were susceptible to imipenem. 

Enterobacteriaceae isolates from cow and goat raw milk also showed high (100; 100%) resistance to novobiocin (100; 100%), and vancomycin (100; 100%), tetracycline (100; 100%), and erythromycin (85.7; 100%). Nalidixic acid (42.9; 50%) and kanamycin (0; 33.3%) demonstrated lower of resistance to isolates from cow and goat milk, respectively. None of the Enterobacteriaceae isolates showed resistance to cefpodoxime and imipenem. Our findings suggest that Enterobacteriaceae from farm environment is resistant to common antibiotics used in huma medicine, hence a health risk to consumers.

As indicated in our study, Enterobacteriaceae from goat and cattle farms showed resistance to novobiocin, one of the effective antibiotics used against Gram-negative/Gram-positive microorganisms [67]. According to Bisacchi and Manchester [68], novobiocin is frequently used as a penicillin replacement in the treatment of penicillin-resistant *S. aureus*. Erythromycin (macrolide) and vancomycin are used for treatment of human campylobacteriosis [69] and serious Gram-positive bacterial infections [70], respectively. It is reported that extended use of antibiotics in food animals creates a conducive environment for the development and diffusion of resistant bacteria [71]. Individuals may attain antimicrobial resistant bacteria via the food chain or contaminated soil, manure, water, and raw milk. 

Although low resistance was displayed to cefpodoxime and imipenem in our study, limited studies have recognized the incidence of carbapenemase (CP)-producing bacteria in food-producing animals and surrounding environment [72]. Even though the incidence of CP microbes in food-producing animals is low, CP bacteria spread from food-producing animals to their derivative products is a risk to consumers and result to severe consequences [73]. According to Iovleva and Doi [74], Carbapenem-resistant Enterobacteriaceae (CRE) is on the rise and a major concern to modern medicine.

Multidrug-resistant resistance was demonstrated among the Enterobacteriaceae isolates from manure, soil, and water. A total of four antibiotic resistant patterns were recorded: NOV-ERY-VAN, NOV-TET-ERY-VAN-KAN, and NAL-NOV-TET-ERY-VAN. NOV-TET-ERY-VAN was the most common pattern among the isolates. *E. coli* isolates displayed three antibiotic resistant patterns: NOV-TET-ERY-VAN, NOV-TET-VAN, and TET-VAN. *E. coli* and *Enterobacter aerogenes* displayed resistance to five out of the eight antimicrobials tested. Multidrug-resistant *E. coli* is a concern to the public health to the fact that it is an indicator of antimicrobial resistance of Gram-negative bacteria [75].

Our study presented six (*n* = 6) different AMR patterns among Enterobacteriaceae isolate from raw milk. Multidrug resistant Enterobacteriaceae in farm environment and raw milk is a food safety risk, since bacterial species in this family are often resistant to most of the antibiotics that are used against them [76]. The development of AMR in bacteria may be caused by horizontal gene transfer that originate from bacteria in the environmenta [77].

### 3.5. Antimicrobial Drug Resistance in Pathogenic Bacteria

Our findings demonstrated *E. coli* O157:H7 and *Salmonella* spp. resistance to erythromycin (7%) and vancomycin (7%). Contrary to our study, Sobur et al. [43], noted in their findings that high *E. coli* O157:H7 resistance to erythromycin (88.9%) and tetracycline (89.4%). *E. coli* O157:H7 and *Salmonella* spp. in our study presented three (*n* = 3) and five (*n* = 5) AMR patterns, respectively. Our findings support the Chang et al. [16] study which demonstrated that dairy cows are reservoirs of antimicrobial resistant *E. coli* O157:H7 and *Salmonella* spp. These pathogens may be transmitted to humans through interaction with animals, contaminated soil, manure, and water, or milk [16]. Antimicrobial use in food-animal farming has been assumed to be a source for the emergence and dissemination of antimicrobial resistant *Salmonella* spp. [78]. In our study, imipenem was effective for both *E. coli* O157:H7 and *Salmonella* spp. and agrees with findings [43].

*L. monocytogenes* isolates in cattle and goat farms demonstrated multidrug resistance to most antibiotics tested, such as cefpodoxime (27.9%), kanamycin (27.9%), tetracycline (25.6%), and nalidixic acid (25.6%). Our results display that *Listeria* spp. displayed the most AMR patterns (*n* = 10). The most common of the 10 patterns were TET-CEF-KAN-NAL, displayed by one soil, and two manure isolates. Of the 10 patterns, one isolate from manure displayed resistance to seven of the eight antimicrobials used: TET-VAN-CEF-NOV-KAN-ERY-NAL. *L. monocytogenes* are generally susceptible to antibiotics that are used for treatment of listeriosis [79] Healthy cattle are reservoirs of *Listeria* spp. and through shedding of feces and can potentially contaminate the soil, water sources, milk, and meats [80]. The movement of animals and farm worker within and between farms could also result to the dispersion of *monocytogenes* in the farm environment [81]. Multidrug resistance of *Listeria* spp. strains has also been detected in food and environmental sources [82]. Since *Listeria* spp. is present in all aspects of the environment and a challenge to control [7], the implementation and application of Good Agricultural Practices (GAPs) and Good Management Practices (GMPs) can mitigate the occurrence of antimicrobial resistant *Listeria* spp. in food animal production systems.

*Enterococcus* spp. from cattle and goat farms showed 32.6%, 30.2%, 30.2%, 7% resistance to vancomycin, novobiocin, erythromycin, and tetracycline, respectively. Although antibiotic resistance was at a lower rate in our study, our findings agree with [83] report that stated vancomycin (98%) and erythromycin (82%) resistance to *Enterococcus* spp. from dairy cattle. *Enterococcus* spp. presented six (*n* = 6) AMR patterns. Our study indicates that *Enterococcus* spp. isolates were resistant to Vancomycin. VRE has previously been isolated from manure contaminated feedlot soils [84] and in cattle fecal samples [85]. According to Foka and Ateba [83], VRE is the most widespread multidrug resistant strain of *Enterococcus* spp. *Enterococcus* spp. resistance to both imipenem and cefpodoxime was lowest at 4.7%. *Enterococcus* spp. have been reported to colonize the guts of cattle and humans [86] and are known to survive in varying environments where they cause serious infections [87]. *Enterococci* spp. are reported to have the potential to transfer their antimicrobial resistant genes to other microbes [88], hence their prevalence in cattle and goat farms is a public health concern. According to Simner et al. [89], occurrence of multidrug resistant *Enterococcus* spp. has been attributed to the widespread use and misappropriation of antimicrobials in animal agriculture. 

Generally, our study demonstrated that Enterobacteriaceae isolates from manure, soil, water, and raw milk were resistant to the same antibiotics to some extent. Overflow of antibiotic-resistant bacteria from the animal farming settings to the neighboring environment is creating a potential public health risk throughout the world [3]. Although the understanding on the spread of AMR within farming environments and from animals to humans is limited, food animals are responsible in the propagation of AMR into the environment [90].

## 4. Materials and Methods

### 4.1. Study Sites and Sample Collection

Two cattle farms (CF1 and CF2) and a goat farm (GF) retained by Tennessee State University (TSU) were selected for this study. The study was approved by Institutional Animal Care and Use Committee (IACUC) at TSU. All norms or standards for protection and animal welfare were observed in this study. CF1 and GF are in the main campus agricultural research center in Davidson County, while CF2 is in Cheatham County, approximately 30 miles away from the main campus farm. A total of 210 environmental samples; manure (M) and soil (S) were aseptically collected and evaluated in our study. Briefly, MCF1 = 35; MCF2 = 35; and MGF = 35; SCF1 = 35; SCF2 = 35; and SGF = 35) samples were collected. Specifically, a sterile spoon was used to collect soil at a depth of 5 cm in triplicates in assigned spots on the farms. There were also two types of water samples (runoff water and drinking water in troughs) collected. Overall, sixty-three (CF1 = 21; CF2 =21; GF = 21) trough drinking water samples were collected. Additionally, a total of 12 (GF = 4; CF1 = 2; CF2= 6) runoff water samples were also collected for microbial analysis. Overflow water was collected when it rained, since only then was there surface runoff. 

Additionally, raw milk samples from lactating cattle (*n* = 35) and kidding goats (*n* = 46) were collected according to USDA-APHIS [91]. Approximately, 50 mL milk was collected from each animal into sterile plastic tubes labelled with farm identification (CF1; CF2; GF). All samples were transported in icebox to the laboratory and stored at –20 °C until assayed. 

### 4.2. Evaluation of Enterobacteriaceae in Cattle and Goat Farms

Soil, water, manure, cow, and goat milk were evaluated for Enterobacteriaceae and for detection, 1 g (solid) or /mL (liquid) of all samples collected was enriched in 9 mL of Difco Enterobacteriaceae enrichment broth Mossel (BD, Sparks, MD, USA) and incubated at 37 °C for 24 h. After enrichment, 1μL of each sample was streaked onto Violet Red Bile Glucose Agar (VRBG)) (Oxoid, Basingstoke, Hants, England) and incubated at 37 °C for 18–24 h. Red to dark purple colonies surrounded by red-purple halos were identified as presumptive Enterobacteriaceae. For further characterization, presumptive colonies were further biochemically characterized using oxidase and API 20E (bioMerieux, Hazelwood, MO, USA) test methods. The API web software was used to identify Enterobacteriaceae, and species identified above the >90% confidence level were recorded and preserved in sterilized 80% glycerol (BD, Franklin Lakes, NJ, USA) at −80 °C for further analysis.

### 4.3. Pre-Enriched to Select Pathogenic Bacteria 

Environmental (soil, manure, water) and milk samples were also collected and enriched specifically for selected pathogenic bacteria. For all samples, 25 g of manure or soil and 25 mL of water and milk for each sample was addended into a stomacher bag (Fisher scientific, Pittsburgh, PA, USA) with 225 mL buffered peptone water (BPW) (Oxoid, Solon, OH, USA). The mixture was then homogenized (Stomacher^®^ 400 Circulator, Seward, Norfolk, UK) at 230 rpm for 2 min and pre-enriched at 37 °C for 24 h.

### 4.4. Detection of E. coli O157:H7

For the detection of *E. coli* O157:H7, 0.1 mLof each pre-enriched sample was added to 10 mL of enterohemorrhagic *E. coli* enrichment broth (BD Franklin Lakes, NJ, USA) and incubated at 37 °C for 24 h. Consequently, a loop (10 μL) of each cultured broth was streaked onto Sorbitol MacConkey agar (CM0813; Oxoid) enhanced with cefixime (50 ng/mL) and potassium tellurite (25 mg/mL) supplement (SR0172E; Oxoid) agar plates and incubated at 37 °C for 24 h. Presumptive colorless colonies were recorded as presumptive *E. coli* O157:H7 isolates.

### 4.5. Detection of Salmonella

Approximately 0.1 mL of each pre-enriched sample was added to 10 mL of Rappaport-Vassiliadis (RV) broth (BD, Franklin Lakes, NJ, USA) and at 37 °C for 24 h. After incubation, a loop (10 μL) of cultured broths were streaked onto Xylose-Lysine-Desoxycholate (XLD) selective agar (Oxoid, Basingstoke, Hants, England). Plates were incubated at 37 °C for 24. *Salmonella* was characterized by red–yellow–black centers.

### 4.6. Detection of Enterococcus spp.

For the detection of *Enterococcus* spp., 0.1 mL of each pre-enriched sample was added to 10 mL Enterococcosel agar (BD, Franklin Lakes, NJ, USA) for *Enterococcus* spp. Plates were incubated at 37 °C for 48 h. Isolates with translucent brownish black to black zones were determined as presumptive colorless *Enterococcus* spp. All isolates for selected bacteria were preserved in sterilized 80% glycerol (BD, Franklin Lakes, NJ, USA) at −80 °C for further investigation.

### 4.7. Detection of Listeria spp. and Listeria monocytogenes

To detect *Listeria* spp. and *Listeria monocytogenes*, 1 mL of each pre-enriched sample was enriched in 9 mL of Listeria enrichment broth base (CM0862 Oxoid, Basingstoke, Hampshire, England), enriched with Listeria selective supplement (SR0141E Oxoid, Basingstoke, Hampshire, England), and incubated at 35 °C for 48 h. After enrichment, 10 μL of the enriched culture was streaked onto Listeria selective agar base oxford formulation (CM0856 Oxoid, Basingstoke, Hants, England) with added Listeria selective supplement. The plates were then incubated for 48 h at 35 °C. Brownish black colonies were identified as presumptive *Listeria* spp. or *L. monocytogenes* colonies which were and subsequently preserved at −80 °C.

### 4.8. PCR Confirmation of Pathogenic and Commensal Bacteria

Presumptive *Escherichia coli* O157:H7, *Salmonella* spp., *Listeria monocytogenes*, *Listeria* spp, and *enterococci* spp. were cultivated overnight at 35 °C in tryptic soy broth (TSB; Difco BD, Franklin Lakes, NJ, USA). DNA was then extracted from the overnight cultures (>5 × 10^6^ cells) by using the UltraClean^®^ Microbial DNA isolation Kit (MO BIO Laboratories, Inc., Carlsbad, CA, USA). DNA concentrations and integrity were then determined by using a NanoDrop 2000 (Thermo Scientific, Pittsburgh, PA, USA) and agarose gel electrophoresis, respectively. Oligonucleotide primer pairs were synthesized (Operon Technologies, Huntsville, AL, USA) to amplify genes of interest. Single PCR was used for *E. coli* O157:H7, *Salmonella* spp., and *Enterococcus* spp. A Hotstar Taq Plus Master Mix (Qiagen, Hilden, Germany) was used in this study. Each 20 µL reaction mixture contained 4 µL DNA template, 1 µL of each primer, 10 µL master mix, 2 µL RNase free water and 2 µL coral load (supplied with the kit). The working concentrations of the primers were 10 ng/µL and 100 ng/µL for the DNA template. *E. coli* O157:H7 (*rfbE*) primer pair was 5′-CAGGTGAAGG TGGAATGGTTGTC-3′ and 5′-TTAGAATTGAGACCATCCAATAAG-3′ [92]. Target gene (*ompC*) for *Salmonella* spp. set of primers was 5′-ATCGCTGACTTATGCAATCG-3” and 5′-CGGGTTGCGTTATAGGTCTG-3′ [93]. Set of primer 5′ -TACTGACAAACCATTCATGATG-3′ and 5′-AACTTCGTCACCAACGCG AAC-3′ targeted *Tuf* gene for *Enterococcus* spp. [94].

A multiplex PCR plus kit (Qiagen, Hilden, Germany) was used for the confirmation of *Listeria* spp. in our samples. Each 50 µL reaction mixture contained 25 µL of master mix, 5 µL of 10× primer mix (2 µM each primer), 100 ng DNA template, 5 µL Q- solution, 5 µL Coral Load dye, and RNase free water. *Listeria* spp. target gene (*prs*) primer pair was 5′-GCTGAAGAGATTGCGAAAGAAG-3′ and 5′-CAAAGAAACCTTGGATTTGCGG-3′ [95]. Target gene (*hly*) for *Listeria monocytogenes* the primer pair was 5′-CATTAGTGGAAAGATGGAATG-3′ and 5′-GTATCCTCCAGAGTGATCGA-3′ [95]. PCR was performed by using a nexus gradient Thermal Cycler (Eppendorf, Hauppauge, NY, USA). PCR products were separated in agarose gel (Fisher Scientific, Fair Lawn, NJ, USA) with TE buffer stained with 0.1 µg/mL of ethidium bromide and photographed under UV light. Water was used as negative control throughout the PCR confirmation. 

### 4.9. Antibiotic Resistance Profiling

Antimicrobial susceptibility testing to 8 antimicrobials was achieved according to the Clinical and Laboratory Standards Institute [96]. Enterobacteriaceae (*n* = 148), *E. coli* O157:H7 (*n* = 3), *Salmonella* spp. (*n* = 29), *Listeria Monocytogenes* (*n* = 66), and *Enterococcus* (*n* = 69) isolates were subjected to the following antibiotics susceptibility disks, with the disk concentration in parentheses: vancomycin (30 μg), novobiocin (30 μg), erythromycin (15 μg), tetracycline (5 μg), cefpodoxime (10 μg), kanamycin (10 μg), nalidixic acid (30 μg), and imipenem (10 μg). Briefly, bacterial cultures were prepared as previously described and were modified to 0.5 McFarland standard and evenly spread on Mueller-Hinton agar plates (Difco, BD). Antibiotic susceptibility disks (BBL, BD) were then applied on Mueller-Hinton agar plates and inhibition zones were examined at 37 °C after 24 h incubation. The results were interpreted based on CLSI for human medicine as resistant or susceptible. *Staphylococcus aureus* ATCC 25923 and *Escherichia coli* ATCC 25922 reference strains and were tested simultaneously as positive controls.

### 4.10. Statistical Analysis

The prevalence and antibiotic resistant profiles for bacteria were analyzed using the analysis of variance of SAS for Windows (version 6.12; SAS Institute, Inc., Cary, NC, USA) and chi-square test. The antibiotic resistance values were expressed as percentages and *p*-value less than 0.05 was considered statistically significant.

## 5. Conclusions

Transfer of AMR from animals to humans and the environment can be transmitted by both pathogenic and commensal bacteria. The findings of this study indicate that *E. coli* O157:H7, *Salmonella* spp. and *Listeria monocytogenes*, as well as opportunistic pathogens such as *Enterococcus* from cattle and goat farms, were resistant to clinically important antibiotics. Resistant bacteria circulating in animal farms threaten both animal and human health. Hence, livestock producers should be sensitized on AMR challenges, alternative choices to using antibiotics, such as improved husbandry practices and hygiene, as well as use of vaccinations. Educated animal producers will make informed decisions which will contribute to mitigation of AMR. Further research in a larger scale is imperative to explore the AMR patterns in small-scale food animal production systems to ensure food safety.

## Figures and Tables

**Table 1:** Prevalence of Enterobacteriaceae (%) in environmental samples from goat and cattle farms.

Bacterial Species	CF1 (*n* = 55)				CF2 (*n* = 46)				GF (*n* = 47)				Total
	MA	SO	TW	RW	MA	SO	TW	RW	MA	SO	TW	RW	
*Citrobacter braakii*	0	0	0	0	1	0	0	0	0	0	0	0	1 (0.7) ^c^
*Chryseomonas luteola*	0	0	0	0	0	0	0	0	0	1	0	0	1 (0.7) ^c^
*Citrobacter freundii*	0	0	0	0	0	0	0	0	0	1	0	0	1 (0.7) ^c^
*E. coli*	25	12	0	5	20	15	1	0	23	8	2	2	113 (76.4) ^a^
*E. fergusonii*	1	0	0	0	0	0	0	0	0	0	0	0	1 (0.7) ^c^
*Enterobacter aerogenes*	0	1	0	0	0	0	0	0	0	1	0	0	3 (2.0) ^c^
*Enterobacter* *amnigenus*	0	0	0	0	0	0	0	0	0	0	1	0	1 (0.7) ^c^
*Enterobacter cloacae*	4	1	0	0	0	4	1	0	1	3	1	0	15 (10.1) ^b^
*Hafnia alvei*	0	1	0	0	0	0	0	0	0	0	0	0	1 (0.7) ^c^
*Klebsiella pneumoniae*	0	0	0	0	1	0	0	0	0	0	0	0	1 (0.7) ^c^
*Kluyvera* sp.	0	1	0	0	0	0	0	0	0	0	0	0	1 (0.7) ^c^
*Serratia marcescens*	0	1	0	0	0	1	1	0	0	1	0	0	5 (3.4) ^c^
*Serratia odorifera*	1	1	0	0	0	0	0	0	0	0	0	0	2 (1.4) ^c^
*Stenotrophomonas maltophilia*	0	0	0	0	0	0	0	0	0	1	0	0	1 (0.7) ^c^
*Yersinia enterocolitica*	0	0	0	0	0	0	0	0	0	0	0	1	1 (0.7) ^c^
Total	31	18	1	5	22	21	3	0	24	16	4	3	148

CF1—Cattle Farm 1; CF2—Cattle Farm 2; GF—Goat Farm; MA—manure; SO—soil; TW—Trough Water; RW—Runoff Water; ^a–c^ Mean values (%) within column with different superscript differ significantly (*p* < 0.05).

**Table 2:** Occurrence of Enterobacteriaceae in raw cow and goat milk.

Enterobacteriaceae	Cow Milk	Goat Milk	Total
	(*n* = 6)	(*n* = 7)	
*Enterobacter amnigenus* 2		1(7.7)	1(7.7)
*Enterobacter cloacae*		1(7.7)	1(7.7)
*Escherichia coli*		3(23.1)	3(23.1)
*Escherichia hermannii*		1(7.7)	1(7.7)
*Klebsiella pneumoniae*		1(7.7)	1(7.7)
*Pantoea* spp. 3	1(7.7)		1(7.7)
*Pantoea* spp. 4	5(38.5)		5(38.5)
Total	6 (46.2) ^a^	7(53.8) ^a^	13 (100)

^a^ Mean values (%) within rows with different superscript differ significantly (*p* < 0.05).

**Table 3:** Distribution of pathogenic bacteria in cattle and goat farms.

Bacteria	CF1				CF2				GF				
	Manure	Soil	W-T	W-R	Manure	Soil	W-T	W-R	Manure	Soil	W-T	W-R	Total
*E. coli* O157:H7	2 (0.7) ^a^	1 (0.4) ^a^	0 ^b^	1 (0.4) ^a^	1 (0.4) ^a^	0 ^b^	0 ^b^	0 ^b^	0 ^b^	0 ^b^	0 ^b^	0 ^b^	5 (1.9) ^z^
*Enterococcus*	7 (2.5) ^bc^	9 (3.2) ^ab^	5 (1.8) ^cd^	3 (1.1) ^d^	10 (3.5)^ab^	8 (2.8) ^abc^	3 (1.1) ^d^	0 ^f^	12 (4.2) ^a^	8 (2.8)^abc^	3 (1.1)^d^	1(0.4) ^a^	69 (24.5) ^x^
*L.monocytogenes*	8 (2.8) ^bc^	8 (2.8) ^bc^	4 (1.4) ^d^	1 (0.4) ^a^	11 (3.9)^ab^	13 (4.6)^a^	2 (0.7) ^de^	0 ^f^	6 (2.1) ^c^	9 (3.2) ^ab^	3 (1.1)^d^	1(0.4) ^a^	66 (23.4) ^x^
*Salmonella*	2 (0.7) ^cd^	3 (1.1) ^bc^	0 ^e^	1 (0.4) ^a^	2 (0.7)^cd^	8 (2.8) ^a^	0 ^e^	1 (0.4)^d^	6 (2.1) ^c^	5 (1.8) ^cd^	1 (0.4) ^a^	0 ^e^	29 (10.4) ^y^

CF1—Cattle Farm 1; CF2—Cattle Farm 2; GF—Goat Farm; TW—Trough Water. RW—Runoff Water, ^a–f^ Mean values (%) within rows with different superscripts differ significantly at (*p* < 0.05). ^x–z^ Mean values (%) within the column with different superscripts differ significantly (*p* < 0.05).

**Table 4:** Antibiotic resistance phenotypes of Enterobacteriaceae isolated from cattle and goat farms.

Bacterial Species	AMR Profile	Number (%) of Isolates		
		CF1	CF2	GF
*Atrobactor brakii*	NOV, TET, ERY, VAN	0 (0) ^c^	1 (0.7) ^b^	0 (0) ^c^
*Chryseomonas luteola*	NOV, TET, ERY, VAN	0 (0) ^c^	0 (0) ^b^	1 (0.7) ^c^
*Citrobacter freundii*	NAL, NOV, TET, ERY, VAN	0 (0) ^c^	0 (0) ^b^	1 (0.7) ^c^
*E. coli*	NOV, ERY, VAN	12 (8.1) ^b^	5 (3.4) ^b^	8 (5.4) ^b^
*E. coli*	NOV, TET, ERY, VAN	30 (20.3) ^a^	32 (21.6) ^a^	25 (16.9) ^a^
*E. coli*	NOV, TET, ERY, VAN, KAN	0 (0) ^c^	0 (0) ^b^	1 (0.7) ^c^
*E. fergusonii*	NOV, TET, ERY, VAN	1 (0.7) ^c^	0 (0) ^b^	0 (0) ^c^
*Enterobacter aerogenes*	NOV, TET, ERY, VAN	1 (0.7) ^c^	1 (0.7) ^b^	0 (0) ^c^
*Enterobacter aerogenes*	NOV, TET, ERY, VAN, KAN	0 (0) ^c^	0 (0) ^b^	1 (0.7) ^c^
*Enterobacter amnigenus* 2	NOV, ERY, VAN	0 (0) ^c^	0 (0) ^b^	1 (0.7) ^c^
*Enterobacter cloacae*	NOV, TET, ERY, VAN	5 (3.4) ^bc^	5 (3.4) ^b^	3 (2.0) ^bc^
*Enterobacter cloacae*	NOV, ERY, VAN	0 (0) ^c^	0 (0) ^b^	2(1.4) ^bc^
*Hafnia alvei*	NOV, ERY, VAN	1 (0.7) ^c^	0 (0) ^b^	0 (0) ^c^
*Klebsiella pneumoniae*	NOV, TET, ERY, VAN	0 (0) ^c^	1 (0.7) ^b^	0 (0) ^c^
*Kluyvera* spp.	NOV, TET, ERY, VAN	1 (0.7) ^c^	0 (0) ^b^	0 (0) ^c^
*Serratia marcascens*	NOV, TET, ERY, VAN	2 (1.4) ^c^	1 (0.7) ^b^	0 (0) ^c^
*Serratia marcascens*	NOV, ERY, VAN	1 (0.7) ^c^	0 (0) ^b^	0 (0) ^c^
*Serratia odorifera*	NOV, TET, ERY, VAN	1 (0.7) ^c^	1 (0.7) ^b^	0 (0) ^c^
*Stenotrophomonas maltophilia*	NOV, TET, ERY, VAN	0 (0) ^c^	0 (0) ^b^	1 (0.7) ^c^
*Yersinia enterocolitica*	NOV, ERY, VAN	0 (0) ^c^	0 (0) ^b^	1 (0.7) ^c^

CF 1—Cattle Farm 1; CF 2—Cattle Farm 2; GF—Goat Farm; NOV—Novobiocin; TET—Tetracycline; ERY—Erythromycin; VAN—Vancomycin; KAN, Kanamycin; NAL—Nalidixic acid. ^a–c^ Mean values (%) within columns with different superscripts differ significantly (*p* < 0.05).

**Table 5:** Antibiotic resistant patterns of Enterobacteriaceae species isolated from cow and goat milk.

Enterobacteriaceae		Number of Isolates	
	AMR Profile	Cow Milk	Goat Milk
*Enterobacter amnigenus* 2	NOV, TET, ERY, VAN	0 ^c^	1 (7.69) ^a^
*Enterobacter cloacae*	NOV, TET, ERY, VAN	0 ^c^	1 (7.69) ^a^
*Escherichia coli*	NOV, TET, VAN	0 ^c^	1 (7.69) ^a^
*Escherichia coli*	NOV, TET, ERY, VAN	0 ^c^	1 (7.69) ^a^
*Escherichia coli*	TET, VAN	0 ^c^	1 (7.69) ^a^
*Escherichia hermannii*	NOV, TET, ERY, VAN	0 ^c^	1 (7.69) ^a^
*Pantoea* spp. 3	NOV, ERY, VAN	1 (7.69) ^b^	0 ^b^
*Pantoea* spp. 4	NOV, TET, ERY, VAN	3 (23.08) ^a^	0 ^b^
*Pantoea* spp.	NOV, VAN	1 (7.69) ^b^	0 ^b^
*Pantoea* spp.	NOV, TET, VAN	1 (7.69) ^b^	0 ^b^

NOV—Novobiocin; TET—Tetracycline; ERY—Erythromycin; VAN—Vancomycin; KAN—Kanamycin; NAL—Nalidixic acid. ^a–c^ Mean values (%) within columns with different superscripts differ significantly at (*p* < 0.05).

**Table 6:** Antibiotic resistant pathogenic bacteria from farm environment.

Bacteria	Tested Isolates	IPM	TET	VAN	CPD	NOV	KAN	ERY	NAL
*E. coli* O157:H7	3	0(0.0) ^ax^	2(4.7) ^ayz^	3(7.0) ^ay^	1(2.3) ^ay^	2(4.7) ^ay^	1(2.3) ^ay^	3(7.0) ^ay^	2(4.7) ^ay^
*Salmonella*	12	0(0.0) ^bx^	5(11.6) ^bxy^	12(27.9) ^ax^	3(7.0) ^by^	12(27.9) ^ax^	1(2.3) ^by^	12(27.9) ^ax^	1(2.3) ^by^
*Enterococcus*	14	2(4.7) ^ax^	3(7.0) ^byz^	14(32.6) ^ax^	2(4.7) ^by^	13(30.2) ^ax^	2(4.7) ^by^	13(30.2) ^ax^	2(4.7) ^by^
*L. monocytogenes*	14	1(2.3) ^bx^	11(25.6) ^ax^	7(16.3) ^axy^	12(27.9) ^ax^	7(16.3) ^bxy^	12(27.9)^ax^	3(7.0) ^by^	11(25.6) ^ax^
Total	43	3 (7.0) ^c^	21 (48.8) ^b^	36 (83.7) ^a^	18 (41.9) ^b^	34 (79.1) ^a^	16 (37.2) ^b^	31 (72.1) ^a^	16 (37.2) ^b^

ERY—Erythromycin; NOV—Novobiocin; CPD—Cefpodoxime; NAL—Nalidixic acid; IPM—Imipenem; KAN—Kanamycin; VAN—Vancomycin; TET—Tetracycline. ^a–c^ Mean values (%) within rows with different superscript differ significantly (*p* < 0.05). ^x–z^ Mean values (%) within columns with different superscripts differ significantly (*p* < 0.05).

**Table 7:** Phenotypic patterns of pathogenic bacteria from cattle and goat farms.

Bacteria	AMR Profile	Number (%) of Isolates		
		CF1	CF2	GF
*E. coli* O157:H7	VAN, NOV, KAN, ERY, NAL	1 (2.3) ^bc^	0 ^b^	0 ^c^
	TET, VAN, ERY, NAL	1 (2.3) ^bc^	0 ^b^	0 ^c^
	TE, VAN, CEF, NOV, ERY	1 (2.3) ^bc^	0 ^b^	0 ^c^
*Salmonella*	VAN, NOV, ERY	0 ^c^	3 (7.0) ^a^	3 (7.0) ^b^
	VAN, CEF, NOV, ERY	0 ^c^	0 ^b^	1 (2.3)
	TET, VAN, CEF, NOV, ERY	1 (2.3) ^bc^	0 ^b^	0 ^c^
	TE, VAN, CEF, NOV, KAN	0 ^c^	1 (2.3) ^ab^	0 ^c^
	ERY, NAL	3 (7.0) ^b^	0 ^b^	0 ^c^
*Enterococcus*	TE, VAN, NOV, ERY	4 (9.3) ^a^	2 (4.7) ^a^	4 (9.3) ^a^
	TET, VAN, NOV, ERY	0 ^c^	0 ^b^	1 (2.3) ^bc^
	IMP, TE, VAN, CEF, KAN, NAL	0 ^c^	1 (2.3) ^ab^	0 ^c^
	IMP, VAN, NOV, ERY	1 (2.3) ^bc^	0 ^b^	0 ^c^
	TE, VAN, CEF, NOV, KAN, ERY, NAL	0 ^c^	1 (2.3) ^ab^	0 ^c^
*L. monocytogenes*	IMP, TET, VAN, CEF, NOV, KAN, ERY, NAL	0 ^c^	0 ^b^	1 (2.3) ^bc^
	KAN	0 ^c^	1 (2.3) ^ab^	0 ^c^
	NOV	1 (2.3) ^bc^	0 ^b^	0 ^c^
	TE, CEF	1 (2.3) ^bc^	0 ^b^	0 ^c^
	TE, CEF, KAN, NAL	1 (2.3) ^bc^	1 (2.3) ^ab^	1 (2.3) ^bc^
	TE, CEF, NOV, KAN, NAL	0 ^c^	0 ^b^	1 (2.3) ^bc^
	TE, VAN, CEF, KAN, NAL	0 ^c^	2 (4.7) ^a^	0 ^c^
	TE, VAN, CEF, NOV, KAN, ERY, NAL	1 (2.3) ^bc^	0 ^b^	0 ^c^
	TE, VAN, CEF, NOV, KAN, NAL	1 (2.3) ^bc^	0 ^b^	1 (2.3) ^bc^
	VAN, CEF, NOV, KAN, NAL	0 ^c^	1 (2.3) ^ab^	1 (2.3) ^bc^
	TE, VAN, NOV, ERY	0 ^c^	0 ^b^	1 (2.3) ^bc^

NOV—Novobiocin; TE—Tetracycline; ERY—Erythromycin; VAN—Vancomycin; KAN—Kanamycin; NAL—Nalidixic acid. ^a–c^ Mean values (%) within columns with different superscripts differ significantly (*p* < 0.05).

## Data Availability

Not applicable.
